# Willingness of health care providers to offer HIV self-testing from specialized HIV care services in the northeast of Brazil

**DOI:** 10.1186/s12913-022-08091-2

**Published:** 2022-05-30

**Authors:** Tiago Jordão, Laio Magno, Marcos Pereira, Thais Regis Aranha Rossi, Pedro de Almeida Silva, Maria Aparecida Araújo Figueiredo, Nília Maria de Brito Lima Prado, Adriano Maia dos Santos, Maria Cristina Cangussu, Inês Dourado

**Affiliations:** 1 Departamento de Ciências da Vida, Universidade do Estado da Bahia, Rua Silveira Martins, 2555, Cabula, Salvador, 41.150-000 Brazil; 2 Diretoria de Vigilância Epidemiológica da Bahia, Secretaria de Saúde do Estado da Bahia, Salvador, Brazil; 3 Instituto de Saúde Coletiva, Universidade Federal da Bahia, Salvador, Brazil; 4 Instituto Multidisciplinar em Saúde, Universidade Federal da Bahia, Vitória da Conquista, Brazil; 5 Faculdade de Odontologia, Universidade Federal da Bahia, Salvador, Brazil

**Keywords:** HIV self-test, Health care providers, Willingness to offer, Brazil

## Abstract

**Background:**

The insufficient knowledge regarding the serological status of people affected with human immunodeficiency virus (HIV) is a concern in Brazil. HIV self-testing (HIVST) has been proved to have great potential for increasing testing, especially among vulnerable populations. The large-scale distribution of HIVST by the Brazilian public health system has increased in recent years. We aimed to investigate the awareness of HIVST among health care providers (HCP) from specialized HIV/AIDS care services in the state of Bahia, Northeast Brazil. Further we investigated HCP acceptability and willingness to offer its use.

**Methods:**

A cross-sectional study on HCP from 29 specialized care services (SCS) located in 21 cities in the state of Bahia. HCP working in the service for at least 6 months were included. Sociodemographic, occupational, and behavioral data were collected using a questionnaire. Descriptive statistics were carried out. Bivariate, and multivariate analyses estimating adjusted odds ratios (aOR) and 95% confidence intervals (95% CI) using logistic regression were conducted.

**Results:**

The awareness and acceptability of HIVST and the willingness to provide it were 79.8, 55.2, and 47.1%, respectively. Few HCP reported that the SCS where they worked dispensed HIVST (3.6%), and 13.5% received some information or training on HIVST. Factors associated with willingness to offer HIVST were: HIVST acceptability (aOR = 9.45; 95% CI: 4.53–19.71), willingness to use HIVST on themselves (aOR = 4.45; 95% CI: 1.62–12.24), confidence in offering HIVST to clients (aOR = 5.73; 95% CI: 2.26–12.72), and considering everyone eligible for HIVST (aOR = 2.88; 95% CI: 1.25–6.59).

**Conclusions:**

Although most HCP were aware of HIVST, acceptability and willingness to provide it to the clients was moderate. The scale up of HIVST as a mean for the HIV prevention and control policy in Brazil, requires further training of HCP and better implementation of this program.

**Supplementary Information:**

The online version contains supplementary material available at 10.1186/s12913-022-08091-2.

## Background

Early human immunodeficiency virus (HIV) diagnosis and treatment are important for enhancing the quality of life of people living with HIV (PLHIV) [1] and for reducing viral transmission [2–4]. Since the Joint United Nations Programme on HIV/AIDS (UNAIDS) set its 90–90-90 target in 2014, early HIV detection among PLHIV has become a priority for the signatory nations [5]. To reach this target, new strategies could be introduced to increase HIV testing uptake and awareness of HIV status, including self-testing [6, 7], especially because late diagnosis remains a significant barrier against this target in Latin America [8]. In Brazil, 88% of people living with HIV who know their status by 2019 (i.e., the first of the 90–90-90 targets) [9]. In 2014, Brazil introduced its “test, treat” initiative, which boosted early treatment, with the acquired immune deficiency syndrome (AIDS) detection rate and AIDS mortality rate in 2015 falling by almost 60 and 73%, respectively, compared to the 2007–2014 rates [10]. However, there is a need for testing among populations at increased risk for HIV that fall outside the reach of health policies, such as sex workers, drug users, gay men and men who have sex with men (MSM), transgender people, and the in prison population and their partners [11].

Thus, HIV self-testing (HIVST) has the potential of increasing testing, especially among key vulnerable populations because they face difficulty in accessing existing testing services, mainly because of HIV-related stigma and fear of discrimination in case of a positive result [12, 13]. The distribution of HIVST in large-scale by the Brazilian National Public Health System (in Portuguese: *Sistema Único de Saúde – SUS*) has increased in recent years. HIVST was provided by SUS in 2018, but only for special cases, such as those of Brazilian Ministry of Health (MoH) pilot projects; until then, there were no national nor local training programs to HIVST for health care providers (HCP), and the MoH guidelines regarding HIVST use and distribution were published only in 2019 [14]. Although HISVT large scale distribution was encouraged by the MoH since the begining of the COVID-19 pandemic, there were no specific instructions regarding proper information from HCP to understand HIVST supposed risks and its potential benefits in a scenario of lockdown. However, since the onset of the COVID-19 pandemic, these restrictions have been eased and HIVST is prioritized in key populations across the entire SUS services, wherever it is available at local health facilities [15]. The MoH considers as key populations, gay and other MSM, transgender people, alcohol and other drug users, prison inmates, and sex workers [16].

HIVST is well-received and its acceptability among users is high, especially among people from various subgroups of key populations in different countries [17–19]. The benefits reported by HCP and HIVST users in low- and middle-income countries from Africa include safety of the home environment, avoiding discrimination at healthcare facilities, confidentiality, time saved from not having to travel, having to wait less for the result, lower cost, increased coverage via peer-distribution when compared with other testing initiatives, user emancipation, serosorting, adoption of safe sexual practices, and secondary peer distribution [20–22]. Furthermore, HIVST users believe that HIVST is easy to use and its results are easy to understand [23, 24].

However, HCP concerns could hamper the distribution of and increased access to HIVST. These concerns include the potential for users to not use the test or read the result correctly, psychosocial risks arising from a positive result, and the absence of counselling [25, 26]. Overcoming such concerns is key to enabling greater availability of HIVST via health systems; however, to the best of our knowledge, no studies have been conducted among HCP in Latin America to understand their conduct and attitudes regarding HIVST. Accordingly, we aimed to investigate HIVST awareness among HCP from specialized HIV/AIDS care services in the state of Bahia. Further we investigated HCP acceptability and willingness to offer its use, and the factors associated.

## Methods

### Study design, sites and population

A cross-sectional study was conducted with HCP from specialized HIV/AIDS care services in Bahia, Northeast Brazil. The HCP working for at least 6 months at the service were included. Specialized care services (SCS) were selected after a single-stage cluster sampling; 25 SCS were selected in the municipalities of Alagoinhas, Barreiras, Bom Jesus da Lapa, Camaçari, Eunápolis, Feira de Santana, Guanambi, Ilhéus, Irecê, Itabuna, Itamaraju, Jequié, Juazeiro, Lauro de Freitas, Paulo Afonso, Porto Seguro, Salvador (with five services), Senhor do Bonfim, Simões Filho, Teixeira de Freitas, and Vitória da Conquista. All HCP available at the services during the data collection period were invited to take part in the study and selected by convenience sampling; 252 HCP at the aforementioned services were recruited from the 490 HCP registered in the National Register of Health Establishments (in Portuguese: *Cadastro Nacional de Estabelecimentos de Saúde*) in March 2020, representing 51.4% of the total population.

The project was assessed and approved by the Research Ethics Committee of the Multidisciplinary Health Institute of the Federal University of Bahia (#3,523,832/2019). Signed informed consent was obtained from all the participants.

### Data collection and instruments

Data collection instrument was a structured questionnaire based on 34 multiple-choice or dichotomous questions, which was developed specifically for this study and administered using tablets in private rooms by trained interviewers. At the end of the interview, the answers were sent to a study database via the internet. The questionnaire was piloted in one of the above-mentioned cities. Data were collected between October 2019 and March 2020.

### Study variables

Awareness of HIVST and its acceptability were dichotomous variables structured according to the following questions: “Did you hear about HIV self-testing before this study?” (no, yes) and “Do you agree with the dispensation of HIV self-tests in this specialized service?” (no, yes). The World Health Organization’s (WHO) HIVST definition was presented to the HCP who responded no to the first question. The outcome variable was willingness to offer HIVST, which was structured according to the question: “Would you offer self-testing for the service client?” (no, yes). Variables that could potentially explain willingness to offer HIVST was selected based on a literature review [17, 20, 27, 28].

The *sociodemographic* variables were: sex (male, female), age (≤ 35, 35 to 50, ≥ 50 years), and education (high school graduate, college graduate, graduate diploma or higher). The *training and occupation* variables were: job (nurse, nursing assistant, physician and others), specialized in HIV/AIDS (no, yes), years of training (≤ 5, 5 to 10, > 10 years), and type of employment contract (temporary, permanent).

The HIVST-related variables were: gives clients information on HIVST (no, yes), sources of previous self-information about HIVST (i.e., how the professional learned about HIVST before the research), reasons for not offering HIVST (suicide risk, self-harm, incorrect use and others), prior training in HIVST (no, yes), HIVST distribution at the service where they work (no, yes), knows that HIVST is available via SUS (no, yes), knows HIVST is available at pharmacies (no, yes), confidence in the HIVST diagnosis (no, yes), willingness to use HIVST on themselves (no, yes), populations they believe HIVST should be distributed in (general public, key populations), sure about offering HIVST to the service users (unsure, quite sure, sure/very sure), believes HIVST results in risk compensation (no, yes), believes HIVST causes a reduction in high-risk sexual behaviors (no, yes), preferred form of dispensing HIVST (assisted testing or self-testing, as per the user), and other resources that should be offered together with HIVST (counselling, prevention materials).

### Data analysis

A descriptive statistics to caracterize awareness, acceptability, and willingness of the HCP to offer HIVST, providing HIVST information to the clients, and the other study variables, estimating the proportions with 95% confidence interval (95% CI). Subsequently, the potential factors associated with willingness to offer HIVST were investigated using bivariate and multivariate analyses, estimating the respective odds ratios (OR) and 95% CI by logistic regression. The variables with *p* <  0.20 in the bivariate analysis, using the chi-squared test, were selected and included in the multivariate analysis. The Hosmer-Lemeshow test (*p* > 0.16) was used to assess the models’ goodness of fit, and the ROC curve estimation (0.88) was used to select the final model and the theoretical relevance of the variables. All data were analyzed using STATA, version 14.0.

## Results

In total, 252 HCP from SCS were interviewed, 78.2% of whom were female and 54.4% were 35–50 years old. The median age of the participants was 43.0 years (interquartile range [IQR]: 37.0–52.0). Most (84.5%) of them had a college degree, around half (51.0%) had a graduate-level diploma, 7.2% had a master’s degree, and 2.4% had a doctorate degree. Regarding their professions, 25.8% were nurses, 12.3% were pharmacists, 11.9% were nursing assistants, 11.9% were physicians, and 9.5% were social workers. Overall, 23.4% had specialized training in HIV/AIDS and over half of them were trained as HCP for more than 10 years (74.2%) (Table 1).

**Table 1:** Sociodemographic, training and occupation characteristics of health care providers in HIV specialized care services in Bahia and Brazil, 2019–2020

Variables	n/N	%	95% CI
**Sociodemographic**			
**Sex**			
Male	55/252	21.8	17.12–27.39
Female	197/252	78.2	72.60–82.87
**Age**			
Age, median (IQR), y.o.	252	43.0^a^	37.0–52.0^b^
Age, proportion			
≤ 35 years old	46/252	18.3	13.93–23.55
35–50 years old	137/252	54.4	48.13–60.45
> 50 years old	69/252	27.4	22.19–33.25
**Education**			
High school graduate	39/251	15.5	11.53–20.60
University graduate	50/251	19.9	15.40–25.36
Graduate diploma	128/251	51.0	447.8–57.17
Residency	10/251	4.0	2.14–7.27
Master’s	18/251	7.2	4.55–11.12
Doctorate	6/251	2.4	1.07–5.24
**Training and Occupation**			
**Job**			
Nurse	65/252	25.8	20.73–31.59
Nursing assistant	30/252	11.9	8.42–16.55
Physician	30/252	11.9	8.42–16.55
Pharmacist	31/252	12.3	8.76–16.99
Social Worker	24/252	9.5	6.44–13.84
Psychologist	15/252	6.0	3.60–9.66
Occupational therapist	2/252	0.8	0.19–3.14
Physiotherapist	2/252	0.8	0.19–3.14
Nutritionist	2/252	0.8	0.19–3.14
Dentist	7/252	2.8	1.32–5.73
Other	44/252	17.5	13.22–22.69
**Specialized in HIV/AIDS**			
Yes	59/252	23.4	18.56–29.07
No	193/252	76.6	70.92–81.43
**Years of training as health provider care**			
≤ 5 years	23/252	9.1	6.12–13.39
5 to 10 years	42/252	16.7	12.53–21.82
> 10 years	187/252	74.2	68.40–79.26
**Type of employment contract**			
Temporary	85/252	33.7	28.12–39.83
Permanent	167/252	66.3	60.16–71.87

^a^Median

^b^Interquartile range

**Fig. 1:**
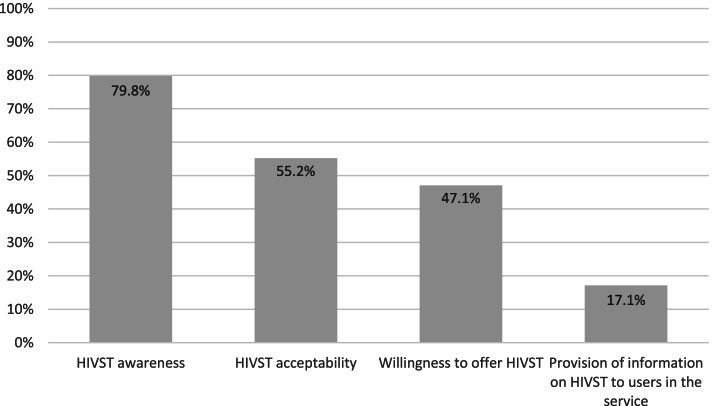
Awareness, acceptability, willingness of HCP to offer HIVST in Bahia and Brazil, 2019–2020

**Fig. 2:**
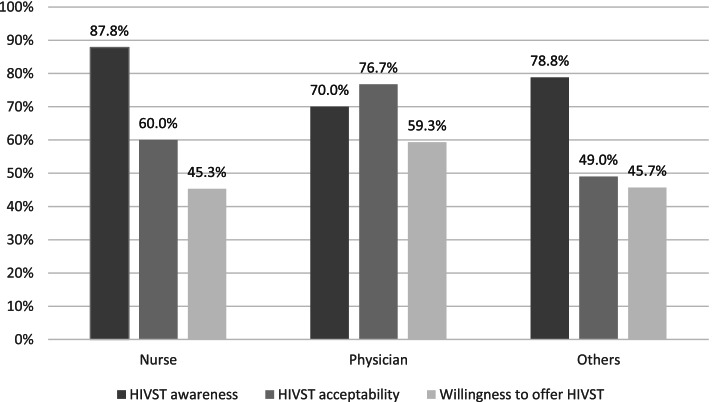
Awareness, acceptability, willingness to offer HIVST by professional category Bahia and Brazil, 2019–2020

Figure 1 shows that 79.8% (95% CI: 74.30–84.30) of the HCP were aware of HIVST, 55.2% (95% CI: 48.92–61.23) agreed with dispensing HIVST at the SCS where they worked, 47.1% (95% CI: 40.90–53.45) were willing to offer HIVST, and 17.1% (95% CI: 12.88–22.25) informed the clients about HIVST. Comparing the results among nursing professions, medical professions, and other professions, the proportion respectively were 87.7, 70.0, and 78.8% for awareness (*p* = 0.10), 60.0, 76.7, and 49.0% for acceptability (*p* = 0.01), and 45.3, 59.3, and 45.7% for willingness to offer HIVST (*p* = 0.40) (Fig. 2).

The main sources of previous information about HIVST were continuing education or training at work (34.5%) and media (TV, radio) (28.4%). The reasons for not offering HIVST were: client suicide risk in case of a positive result (75.4%), failure to use the test or read the result correctly (68.4%), risk of self-harm or harm to others in case of a positive result (61.5%), need for post-test counselling even for a negative test result (55.0%), not knowing where to seek help in the case of a positive test result (50.4%), high risk of test results being discovered by cohabitants or family members (30.5%), and being forced to take the test before sexual intercourse (25.2%) (Table 2).

**Table 2:** Self-information sources about HIVST and reasons for not offering HIVST, Bahia and Brazil, 2019–2020

Variables	n/N	%	IC95%
**Sources of previous self-information about HIVST**			
In-house training	68/197	34.5	28.15–41.48
Media (TV, radio, other)	56/197	28.4	22.51–35.18
Internet or social media	26/197	13.2	9.11–18.73
Coworkers	23/197	11.7	7.85–17.01
Other	24/197	12.2	8.27–17.58
**Reasons for not offering HIVST**			
Suicide risk in case of positive result	101/129	75.4	67.27–82.00
Failure to use the test or read the result correctly	91/129	68.4	59.93–75.83
Risk of self-harm or harm to others in case of positive result	80/129	61.5	52.79–69.59
Need for post-test counselling even for a negative test result	71/129	55.0	46.27–63.50
Not knowing where to seek care in case of a positive result	66/129	50.4	41.78–58.95
High risk of test results being discovered by cohabitants or family members	40/129	30.5	23.17–39.04
Being forced take the test before sexual intercourse	33/129	25.2	18.41–33.43
Other	21/129	16.3	10.79–23.79
**General aspects of HIVST**			
**HIVST distribution at the service where they work**			
Yes	9/252	3.6	1.86–6.74
No	243/252	96.4	93.25–98.13
**Received training on HIVST**			
Yes	34/252	13.5	9.77–18.32
No	218/252	86.5	81.67–90.22
**Knows that HIVST is available via SUS**			
Yes	58/252	23.0	18.20–28.65
No	194/252	77.0	71.34–81.79
**Knows that HIVST is available at pharmacies**			
Yes	156/252	61.9	55.71–67.73
No	96/252	38.1	32.26–44.28
**Confidence in the HIVST diagnosis**			
Yes	140/252	55.6	49.32–61.61
No	112/252	44.4	38.38–50.67
**Willingness to use HIVST on themselves**			
Yes	184/244	75.4	69.57–80.44
No	60/244	24.6	19.55–30.42
**Confidence in offering HIVST to clients**			
Unconfident	96/252	38.1	32.26–44.28
Quite confident	72/252	28.6	23.29–34.49
Confident / very confident	84/252	33.3	27.75–39.42
**Believes HIVST causes risk compensation**			
Yes	108/252	42.9	36.84–49.08
No	144/252	57.1	50.91–63.15
**Believes that access to HIVST reduces high-risk sexual behavior**			
Yes	70/252	28.7	23.32–34.72
No	174/252	71.3	65.27–76.67
**Preferred form of dispensing HIVST**			
Assisted testing at health facility	143/252	56.8	50.51–62.77
Self-testing at venue chosen by user	90/252	35.7	30.00–41.86
As the user wishes	19/252	7.5	4.84–11.54
**Believing HIVST should be dispensed to the public in general**			
Yes	179/248	72.2	66.23–77.43
No	69/248	27.8	22.56–33.76
**Other resources that should be provided together with HIVST**			
Counselling with a focus on HIV and other STIs	172/207	83.1	77.30–87.63
Prevention materials	129/206	62.6	55.75–69.01
Linkage to other strategies	107/203	52.7	45.77–59.54
Materials the user prefers	50/202	24.8	19.24–31.22
Only client registration	21/199	10.6	6.95–15.69

Few HCP reported that the SCS where they worked dispensed HIVST (3.6%), and 13.5% said they received some information or training on HIVST. Only 23.0% of the HCP were aware that HIVST was available via SUS; 61.9% knew it could be purchased at a pharmacy. Slightly over half (55.6%) of the respondents expressed confidence in the diagnosis of HIVST, and 75.4% reported willingness to use HIVST on themselves. Regarding their confidence about offering HIVST, 38.1% said they felt unconfident, 28.6% felt quite confident, and 33.3% felt confident or very confident. Almost half (42.9%) of them believed that HIVST could result in risk compensation, while 28.7% said they believed access to HIVST could reduce high-risk sexual behaviors. Over half (56.8%) of the HCP believed that assisted testing at the health facility was the best option (Table 2).

Regarding the populations eligible for receiving HIVST, 57.1% of the respondents answered that it should be available to everyone, 15.9% believed it should only be available to key populations, and 27.0% believed that both groups should receive it. The other resources or materials used or dispensed together with HIVST were: counselling, focussing on HIV and other sexually transmitted infections (STI) (83.1%); prevention materials (62.6%); linkage to other services, such as HIV Post-Exposure Prophylaxis (PEP) and Pre-exposure prophylaxis (PrEP) (52.7%), materials the client prefers (24.8%); and only client registration (10.6%) (Table 2).

In the bivariate analysis, the factors that positively associated with willingness to offer HIVST were: acceptability of HIVST (OR = 17.22; 95% CI: 8.85–33.49), working at a service where HIVST is dispensed (OR = 9.57; 95% CI: 1.17–77.73), knowing that HIVST is available via SUS (OR = 2.34; 95% CI: 1.27–4.31), confidence in the HIVST diagnosis (OR = 4.19; 95% CI: 2.43–7.23), notifying service users about HIVST (OR = 6.61; 95% CI: 2.91–14.99), willingness to use HIVST on themselves (OR = 10.75; 95% CI: 4.64–24.94), confidence in offering HIVST to clients (confident or very confident; OR = 12.87; 95% CI: 6.21–6.25), believing access to HIVST diminishes high-risk sexual behavior (OR = 2.45; 95% CI: 1.38–4.34), using the test wherever the client prefers (OR = 2.83; 95% CI: 1.63–4.92), and considering everyone eligible for HIVST (OR = 5.36; 95% CI: 2.77–10.36). Regarding the resources that should be dispensed together with HIVST, a positive association was estimated with only client registration (OR = 4.97; 95% CI: 1.60–15.37) and the materials the clients prefers (OR = 3.5; 95% CI: 1.71–7.15) (Table 3).

**Table 3:** Bivariate analysis of willingness to offer HIVST with sociodemographic, training and occupation characteristics, and general aspects of HIVST

Variables	Willingness to offer HIVST		OR	95% CI	*Ρ*-value
	No	Yes			
**Sociodemographic**					
**Sex**					0.27
Male	46.2	53.9	1		
Female	54.7	45.3	0.71	0.38–1.31	
**Age**					0.91
≤ 35 y.o.	51.2	48.8	1		
35–50 y.o.	54.1	45.9	0.88	0.44–1.76	
> 50 y.o.	51.5	48.5	0.98	0.46–2.12	
**Education**					0.75
High school graduate	55.3	44.7	1		
University graduate	44.0	56.0	1.57	0.67–3.67	
Graduate diploma	55.1	44.9	1.00	0.49–2.05	
**Training and Occupation**					
**Specialized in HIV/AIDS**					0.16
No	55.3	44.7	1		
Yes	44.6	55.4	1.53	0.84–2.79	
**Years of training**					0.10
≤ 5 years	36.4	63.6	1		
> 5 years	54.5	45.5	0.47	0.19–1.18	
**Type of employment contract**					0.15
Temporary	46.3	53.7	1		
Permanent	56.2	43.8	0.67	0.39–1.14	
**Knowledge of HIVST**					0.71
No	55.3	44.7	1		
Yes	52.3	47.7	1.12	0.59–2.14	
**Acceptability of HIVST**					< 0.001
No	86.1	13.9	1		
Yes	26.5	73.5	17.22	8.85–33.49	
**General aspects of HIVST**					
**HIVST dispensed at service**					0.01
No	54.5	45.5	1		
Yes	11.1	88.9	9.57	1.17–77.73	
**Received training on HIVST**					0.06
No	55.2	44.8	1		
Yes	38.2	61.8	1.99	0.94–4.19	
**Knows that HIVST is available via SUS**					< 0.01
No	57.8	42.2	1		
Yes	36.8	63.2	2.34	1.27–4.31	
**Knows that HIVST is available at pharmacies**					0.34
No	48.9	51.1	1		
Yes	55.2	44.8	0.77	0.46–1.30	
**Confidence in the HIVST diagnosis**					< 0.001
No	72	28	1		
Yes	38	62	4.19	2.43–7.23	
**Provides service users with information about HIVST**					< 0.001
No	60.2	39.8	1		
Yes	18.6	81.4	6.61	2.91–14.99	
**Willingness to use HIVST on themselves**					< 0.001
No	88.3	11.7	1		
Yes	41.3	58.7	10.75	4.64–24.94	
**Confidence in offering HIVST to clients**					< 0.001
Unconfident	80.2	19.8	1		
Quite confident	51.4	48.6	3.83	1.90–7.68	
Confident / very confident	24.1	75.9	12.87	6.21–6.25	
**Believes that access to HIVST reduces high-risk sexual behavior**					< 0.01
No	59.2	40.8	1		
Yes	37.1	62.9	2.45	1.38–4.34	
**Preferred form of dispensing HIVST**					0.001
Assisted testing at health facility	62.6	37.4	1		
Self-testing at venue chosen by the user	37.1	62.9	2.83	1.63–4.92	
As the user wishes	56.3	43.8	1.30	0.45–3.70	
**Considering everyone eligible for HIVST**					< 0.001
No	79.7	20.3	1		
Yes	42.3	57.7	5.36	2.77–10.36	
**Resources that should be provided together with HIVST**					
**Counselling**					0.08
No	37.1	62.9	1		
Yes	53.1	47	0.52	0.24–1.10	
**Prevention materials**					0.84
No	49.4	50.7	1		
Yes	50.8	49.2	0.94	0.53–1.66	
**Other strategies (PEP, PrEP, etc.)**					0.06
No	57.3	42.7	1		
Yes	43.7	56.3	1.72	0.98–3.03	
**Only client registration**					< 0.02
No	53.9	46.1	1		
Yes	19.1	80.9	4.97	1.60–15.37	
**Materials the user prefers**					< 0.01
No	57.2	42.8	1		
Yes	27.7	72.3	3.50	1.71–7.15	

In the multivariate analysis, a positive association was estimated between willingness to offer HIVST and the following factors: acceptability (adjusted OR [aOR] = 9.45; 95% CI: 4.53–19.71); willingness to use HIVST on themselves (aOR = 4.45; 95% CI: 1.62–12.24); being quite confident about offering HIVST (aOR = 3.09; 95% CI: 1.30–7.31), and being confident or very confident about offering it (aOR = 5.73; 95% CI: 2.26–12.72), compared to unconfident about offering it; and considering everyone eligible for HIVST (aOR = 2.88; 95% CI: 1.25–6.59) (Table 4).

**Table 4:** Multivariate analysis of factors associated with willingness to offer HIVST among HCP, 2019–2020

Variables	aOR	95% CI	*Ρ*-value
**Acceptability of HIVST**			
No	1		
Yes	9.45	4.53–19.71	< 0.001
**Willingness to use HIVST on themselves**			
No	1		
Yes	4.45	1.62–12.24	< 0.01
**Confidence in offering HIVST to clients**			
Unconfident	1		
Quite confident	3.09	1.30–7.31	0.01
Confident /very confident	5.73	2.26–12.72	< 0.001
**Considering everyone eligible for HIVST**			
No	1		
Yes	2.88	1.25–6.59	0.01

## Discussion

This is the first study on HCP from SCS in northeastern Brazil that examined their awareness, acceptability, and willingness to offer HIVST. However, there are several studies investigating these in other populations.

Although most participants enrolled in the study were aware of HIVST (79.8%), their acceptability (55.2%) and willingness to offer it (47.1%) were moderate. It is important for the HCP to perform their relevant role in the HIVST strategy because this is the second most preferred testing modality by clients in several countries [29].

To the best of our knowledge, there are no studies that measured these outcomes among HCP. However, there are several studies investigating these in other populations. In a systematic review of key populations, eight of the 14 studies found high acceptability (≥ 67%), five found moderate acceptability (34 to 66%), and one found low acceptability (≤ 33%) of HIVST [17]. We found moderate acceptability of HIVST among HCP, which differs from the reports of systematic reviews showing high acceptability among specific populations and age groups in different parts of the world [17, 21, 30]. In Brazil, a study conducted in 2016 with MSM 18 years or older in 12 cities, found a similar level of acceptability of HIVST (47.3%), but it was even lower among MSM who never took an HIV test (42.7%) [31]. Although this study did not involve HCP, the coincidental levels may indicate the incipient implementation of self-testing in this country.

In our study, assisted testing at the health facility was the preferred means of administering HIVST; this could indicate that the HCP interviewed could be operating from the traditional perspective of voluntary counselling and testing (VCT), which assumes that clients voluntarily seek services. This approach is different from provider-initiated counselling and testing (PITC), which is marked by the routine proactive testing by HCP at all consultations. Both assume a particular position on the part of the HCP in presenting the client the opportunity to get an HIV test [32, 33]. Considering the Brazilian MoH current proposal to increase HIV testing using HIVST and promote user autonomy, the traditional approach of VCT could hinder the HIVST kit’s distribution, while approaches more akin to PITC could help generate demand for testing.

In this study, less than half of the HCP demonstrated the willingness to offer HIVST. The main reasons for this finding was the potentially negative mental health outcomes, such as suicide risk, self-harm, and harm to others in response to a positive test result. Studies on key populations such as MSM, transgender people, and sex workers in different countries found similar misconceptions ideias about HIVST use, but to date, there is no evidence in the literature that HIVST is associated with such outcomes [34, 35].

The traditional format of counselling prevails among HCP because the absence of post-test counselling for negative test results was also considered a reason for not offering HIVST. In a systematic review of qualitative data collected between 1998 and 2018, Njau et al. [35] found a similar concern among HCP in five African countries. A qualitative study conducted in an AIDS and other STI Counselling and Testing Center in the capital city of Maceió, Brazil, in 2017, found that some HCP believed HIVST could diminish their professional role and could threaten their work, potentially hampering the continuity of care dispensed to cients [36].

In this context, the possibility of not offering HIVST due to lack of counselling could also reduce the capacity to diagnose HIV infection among stigmatized key populations who face multiple barriers in accessing healthcare. Previous experience shows that unconventional testing and counselling formats, such as approaches using online technologies, are related to enhanced HIVST acceptability or enhanced experience of self-testing by users [37–39]. In a 2015–2016 study in the Brazilian capital city of Curitiba, De Boni et al. [40] demonstrated the feasibility of internet-based strategies for the free, anonymous provision of HIVST and information on its use. The repeated use of HIVST in the absence of a HCP but with health care services available to the public is deemed advantageous because it helps in choosing the testing method, increases confidentiality and privacy, and reduces the chance of their suffering from of HIV-related stigma or discrimination [41–44]. Quicker approaches mediated by easy-to-access digital technologies, or options that may not even require real-time user-professional interaction should be considered in future, provided user access is enabled to prevent the risk of excluding the key populations among whom the epidemic is disproportionately high [43, 45]. Therefore, the lack of direct counseling through HCP may be a limitation against new counseling strategies using online tools, which can promote greater test user autonomy [46].

HIVST awareness among HCP was not associated with increased willingness to offer it to users, which could suggest that such willingness maybe influenced by other factors. Conversely, indicators that suggest greater familiarity with HIVST, such as working at a service where it is provided, having received training in HIVST, and knowing about its distribution by SUS are important in increasing the willingness to offer it; thus, there is a need for a broader repertoire of awareness-raising options.

We also found that willingness to offer HIVST was strongly associated with its acceptability. In a systematic review, Sekhon et al. [47] proposed a definition of acceptability that involves a subjective evaluation of the health intervention by both the individual who delivers the intervention and the individual who receives it. These aspects are based on prior knowledge and on prior practical experience of the intervention. The level of specific training in a new health care technology could be a critical factor influencing its uptake [48, 49].

In our study, the HCP who reported willingness to use HIVST on themselves were four times more likely to offer it to clients than those who said they would not use it. This could be a good indicator of confidence in the test and its use. Multi-center studies in Kenya in 2009 and 2010 [50] and in Ethiopia in 2012 [27] with HCP reported that HIVST training and experience using HIVST, respectively, were associated with the willingness to offer it to users.

Willingness to offer HIVST was also higher among those who felt confident about offering it. Around 68% of HCP reported the potential failure of clients to use the test or read the result correctly as a reason for not offering HIVST, which shows their concern regarding laypersons performing the test. The same concerns have also been reported elsewhere [24, 51, 52]. Although errors in conducting the finger-prick test are among the concerns relating to HIVST [53], recent tests indicate that clients and HCP may have the same performance in HIV rapid diagnostic tests [54], and that the finger-prick test is more precise than a saliva test, because the former involves analyzing a blood sample [45].

Confidence in relation to HIVST may be related to the concern with the health service to support clients who present a positive test result. For example, in a systematic review of factors that help and hamper the distribution of HIVST, Musheke et al. [28] identified studies showing that lack of trust in health systems could be a barrier to HIV testing and treatment.

Additionally, our study results indicate that willingness to offer HIVST is high among HCP who agree that everyone should be eligible to receive the HIVST. This indicates that although the vulnerability of certain social groups and the importance of their access to HIVST is recognized, the test could be well-accepted among HCP for large-scale distribution in a comprehensive health system such as the SUS. However, the Brazilian MoH [15] guidelines have focused on the provision of HIVST through strategies towards people using PrEP, people whose sexual partner is living with HIV, and key and priority populations for the HIV epidemic in Brazil [14].

In this scenario, the development of a web-based continuing education on HIVST with focus on resolving doubts and misconceptions, and increasing the confidence of HCP to offer this technology to clients from the HIV SCS, could be an important strategy to increase the acceptability of HIVST and the willingness to offer it. Digital training initiatives have also been used successfully, and their use has helped increase HIVST acceptability and uptake [55, 56]. Therefore, the training based on digital platforms and smart phone applications can be a powerful resource to quickly facilitate access to information about HIVST among HCP.

Owing to the risk of transmission during the COVID-19 pandemic, accessing healthcare facilities was difficult [57, 58]. To continue the HIV testing strategy, the Brazilian MoH encouraged HIVST distribution and use [15]. The lockdown in the cities because the pandemic demanded an effective response regarding HIV screening; however, it could expose people to COVID-19. Thus, HIVST was used to continue HIV testing service during this period because the health facilities were unavailable, representing a break from the traditional health care scenario. This initiative is expected to continue in SUS because there is still a potential risk of new variants of the coronavirus, and it is important to establish new health care approaches, such as telemedicine and other internet-based strategies [59], to avoid interruptions in testing and HIV prevention and care.

The limitations of this study include its sampling process, which was not probabilistic, and the willingness to offer HIVST was analyzed by HCP from SCS that dispensed and did not dispense HIVST. The willigness to offer HIVST among HCP working at services where HIVST was higher. To overcome the above limitations, the municipalities with SCS were randomly selected, considering the nine health districts in the state of Bahia and only one center wherein HIVST was offered was included in the study. Furthermore, it is important to consider that face-to-face interviews between interviewers and may have influenced acceptable responses. To overcome this, the interviewers were selected only if they had no direct link with the services and were previously trained to guarantee the confidentiality of the interview.

## Conclusions

We found a high proportion of HCP aware of HIVST but only a moderate proportion of them reported acceptability and willingness to offer it to clients. Furthermore, gaps in knowledge about HIVST were identified and considered as reasons for not offering it; these included distorted beliefs about HIVST, including suicide risk, self-harm, or harm to others, and the idea that HIVST use could be related to high-risk sexual behaviors. There was a tendency to perpetuate health care practices that reinforce the practices of the HCP and limit individuals’ autonomy regarding their choice of testing.

The large-scale implementation and distribution of HIVST, as is intended for Brazil, depend on investment in training HCP who interact directly with the public to be benefitted by this strategy. The concerns regarding this testing strategy need to be addressed in order to increase the HIV-testing services and a means for the HIV prevention and control. Qualitative studies could help develop a more in-depth understanding of the conceptions of professionals about HIVST and serve as a basis for future interventions, such as training programs focusing on HIVST.

Finally, it is believed that HIVST distribution should be accompanied by structural improvements to assure simple and clear information availability regarding the use of HIVST; this will enable its correct usage and the results would also be read and interpreted correctly, thereby facilitating treatment access for PLHIV.

## Supplementary Information

**Additional file 1.** Questionnaire.

## Data Availability

The data that support the findings of this study are available at “dataverse.harvard.edu”: https://dataverse.harvard.edu/privateurl.xhtml?token=bb9c73db-6801-41c1-965c-eb9621ce2daa.

## Abbreviations

aOR: Adjusted odds ratios
HIVST: HIV self-testing
HCP: Health care providers
SCS: Specialized care services
95% CI: 95% confidence intervals
PLHIV: People living with HIV
SUS: Brazilian public health system
MoH: Brazilian Ministry of Health
VCT: Voluntary counselling and testing
PITC: Provider-initiated counselling and testing
WHO: World Health Organization
