# Supplementary material for: Willingness of health care providers to offer HIV self-testing from specialized HIV care services in the northeast of Brazil
Source: BMC Health Serv Res. 2022 May 30;22:713. doi: 10.1186/s12913-022-08091-2 (PMC9149328; doi:10.1186/s12913-022-08091-2)
Supplement: Supplementary file 1 — Additional file 1. Questionnaire. [file 12913_2022_8091_MOESM1_ESM.docx]

| **QUESTIONNAIRE**  **Willingness to offer HIV self-testing among health care providers from specialized HIV care services in the Northeast of Brazil**  Structured questionnaire was developed for this study. | | | | | | | | |  |
| --- | --- | --- | --- | --- | --- | --- | --- | --- | --- |
| **III.1 - Identificação Geral** | | | | | | | | |  |
| SUP | | Supervisor number: | |  | | number | |  |  |
| ENTREV | | Interviewer number: | |  | | number | |  |  |
| HORIN | | Start time: | |  | | hh:mm:ss | |  |  |
| **III.2 - Identificação do profissional** | | | | | | | | |  |
| SEX | | 1. Sex | |  | | Male | |  |  |
|  |  |  |  |  | | Female | |  |  |
| INSTR | | 1. What is your level of education? | |  | | Complete high school level | |  |  |
|  |  |  |  |  | | Colledge level | |  |  |
|  |  |  |  |  | | Graduate (specialization) | |  |  |
|  |  |  |  |  | | Graduate ( residence ) | |  |  |
|  |  |  |  |  | | Master's degree | |  |  |
|  |  |  |  |  | | Doctorate degree | |  |  |
| FORM | | 1. What is your academic background? | |  | | Nurse | |  |  |
|  |  |  |  |  | | Nursing Technician | |  |  |
|  |  |  |  |  | | Doctor | |  |  |
|  |  |  |  |  | | Psychologist | |  |  |
|  |  |  |  |  | | Pharmaceutical | |  |  |
|  |  |  |  |  | | Occupational Therapist | | |  |
|  |  |  |  |  | | Physiotherapist | | |  |
|  |  |  |  |  | | Nutritionist | |  |  |
|  |  |  |  |  | | Dentist | |  |  |
|  |  |  |  |  | | Social worker | |  |  |
|  |  |  |  |  | | Others | |  |  |
| FORM_001 | | 3.1 Which? (This question only opens when the answer is “11. Other.”) | |  | |  | |  |  |
| ESPECIA | | 1. Do you have a specialty in the area of HIV/AIDS and other STIs? | |  | | Yes | |  |  |
|  |  |  |  |  | | No | |  |  |
| IDAD | | 1. How old are you? | |  | | _______________Years | |  |  |
| ANOFOR | | 1. Years of training as a health professional | |  | | ___________________ | |  |  |
| TRASAE | | 1. When did you start work on the HIV/AIDS SAE? | |  | | _____________________ | |  |  |
| TVINCUL | | 1. In this service you have: | |  | | Temporary contract | |  |  |
|  |  |  |  |  |  | Temporary tender | |  |  |
|  |  |  |  |  |  | Effective tender | |  |  |
| **III.5 -**  **Acceptability and willingness to offer HIVST**  **Explain: The World Health Organization (WHO) defines HIVST as a process in which a person collects their own sample (oral fluid or blood) and then performs a test and interprets the result, alone or with someone they trust .** | | | | | | | | | |
| AUTOTES | | 1. Have you heard of HIVST for HIV before this study? | |  | | Yes | | |  |
|  |  |  |  |  | | No | | |  |
| ONDTES | | 1. Where did you meet or hear about HIVST? | |  | | In continuing education/health service training actions | | |  |
|  |  |  |  |  | | In media (TV, radio and others) | | |  |
|  |  |  |  |  | | On the internet or social network | | |  |
|  |  |  |  |  | | Through co-workers | | |  |
|  |  |  |  |  | | Others | | |  |
| ONDTES_001 | | 10.1 Specify: (This question only opens when the answer is “5. Others) | |  | |  | | |  |
| INAUTES | | 1. Have you received any information material, courses or updates on HIVST ? | |  | | Yes | | |  |
|  |  |  |  |  | | No | | |  |
| OFAUTES | | 1. Are you aware of the offer of HIVST by the public health network? | |  | | Yes | | |  |
|  |  |  |  |  | | No | | |  |
| AUTETFAR | | 1. Are you aware that HIVST can be purchased in private pharmacies? | |  | | Yes | | |  |
|  |  |  |  |  | | No | | |  |
| ACAUTEST | | 1. Do you agree with the dispensation of HIVST for HIV in this specialized service? | |  | | Yes | | |  |
|  |  |  |  |  | | No | | |  |
| INDTEST | | 1. Would you recommend HIVST to users of the service? | |  | | Yes (SKIP TO 21) | | |  |
|  |  |  |  |  | | No | | |  |
| NAUTOTES | | 1. If not, specify the cause of the denial (Multiple-choice answer) | |  | | Suicide risk in case of positive result | | |  |
|  |  |  |  |  | | People with positive results can attack themselves or other people | | |  |
|  |  |  |  |  | | Possible application errors and result reading by users | | |  |
|  |  |  |  |  | | People would not know where to look for care in case of positive results | | |  |
|  |  |  |  |  | | High risk of leaking results information | | |  |
|  |  |  |  |  | | People may be required to get tested before having sex and show the result. | | |  |
|  |  |  |  |  | | It is essential to offer post-test counseling, even in the case of negative results. | | |  |
|  |  |  |  |  | | Another movive | | |  |
|  |  |  |  |  | | 98. Not applicable | | |  |
| NAUTOTES_001 | | **16.1** Which? (This question only opens when the answer is “8. Another reason.”) | |  | |  | | |  |
| EFAUTO | | 1. Is HIVST for HIV efficient in your diagnosis for you? | |  | | Yes | | |  |
|  |  |  |  |  | | No | | |  |
| INAUTO | | 1. Do you inform users of your service about the existence of HIVST ? | |  | | Yes | | |  |
|  |  |  |  |  | | No | | |  |
| USOTEST | | 1. Would you use an HIVST for HIV on yourself? | |  | | Yes (SKIP TO 21) | | |  |
|  |  |  |  |  | | No | | |  |
| PQNUSO | | 1. If not, specify the cause of the denial (Multiple-choice answer) | |  | | Risk of my colleagues knowing the result | | |  |
|  |  |  |  |  | | I prefer other testing modalities | | |  |
|  |  |  |  |  | | I don't trust the effectiveness of HIVST | | |  |
|  |  |  |  |  | | I'm afraid of dealing with a possible positive result alone (o) | | |  |
|  |  |  |  |  | | Other | | |  |
| PQNUSO_001 | | **20.1** Which? (This question only opens when the answer is “5. Other) | |  | |  | | |  |
| INDAUTO | | 1. In your opinion, HIVST should be made available to:   (Multiple choice answer) | |  | | General public | | |  |
|  |  |  |  |  | | Gays and MSM | | |  |
|  |  |  |  |  | | Trans people or *Travestis* | | |  |
|  |  |  |  |  | | Alcohol and/or other drug users | | |  |
|  |  |  |  |  | | sex workers | | |  |
|  |  |  |  |  | | People in the immunological window period | | |  |
|  |  |  |  |  | | PEP or PrEP users | | |  |
| SEGAUTO | | 1. How confidence do you feel about recommending the HIVST kit to users of your service? | |  | | Unconfident | | |  |
|  |  |  |  |  | | Quite confident | | |  |
|  |  |  |  |  | | Confident | | |  |
|  |  |  |  |  | | Very confident | | |  |
| DISAUTRIS | | 1. Do you believe that the availability of access to the HIV/AIDS kit increases high-risk sexual behavior? | |  | | Yes | | |  |
|  |  |  |  |  | | No | | |  |
| DISBAIRIS | | 1. Do you believe that providing access to HIVST can influence the reduction of high-risk sexual behavior? | |  | | Yes | | |  |
|  |  |  |  |  | | No | | |  |
| DISPAUTO | | 1. Does your service dispense HIVST for people? | |  | | Yes | | |  |
|  |  |  |  |  | | No (Skip to 27) | | |  |
| DISPMAT | | 1. If your service does not require HIVST , does your workplace have sufficient availability of materials (self-testing kits) to meet the demands of users? | |  | | Yes | | |  |
|  |  |  |  |  | | No | | |  |
|  |  |  |  |  | | Not applicable | | |  |
| FORMPREF | | 1. In your opinion, what would be the most effective way for the user to use HIVST ? | |  | | I prefer the user to perform the HIVST under the supervision of a professional on the team | | |  |
|  |  |  |  |  | | I prefer the user to choose the location of their choice to perform HIVST | | |  |
| CRECAUT | | 1. What other resources/inputs should be used/dispensed in conjunction with HIVST? (multiple choice question) | |  | | Counseling with a focus on HIV/STI's | | |  |
|  |  |  |  |  | | Registration information only | | |  |
|  |  |  |  |  | | prevention inputs | | |  |
|  |  |  |  |  | | Linking to other strategies (PeP, Prep, etc.) | | |  |
|  |  |  |  |  | | According to user preference | | |  |
|  |  |  |  |  | | Information about the services available to start antiretroviral treatment | | |  |
|  |  |  |  |  | | None | | |  |
